# Gram-Stain Plus MALDI-TOF MS (Matrix-Assisted Laser Desorption Ionization-Time of Flight Mass Spectrometry) for a Rapid Diagnosis of Urinary Tract Infection

**DOI:** 10.1371/journal.pone.0086915

**Published:** 2014-01-22

**Authors:** Almudena Burillo, Belén Rodríguez-Sánchez, Ana Ramiro, Emilia Cercenado, Marta Rodríguez-Créixems, Emilio Bouza

**Affiliations:** 1 Department of Clinical Microbiology & Infectious Diseases, Hospital General Universitario Gregorio Marañón - Instituto de Investigación Sanitaria Gregorio Marañón, Madrid, Madrid, Spain; 2 Facultad de Medicina, Universidad Complutense de Madrid (UCM), Madrid, Madrid, Spain; 3 Red Española de Investigación en Patología Infecciosa (REIPI RD06/0008/1025), Sevilla, Sevilla, Spain; 4 CIBER de Enfermedades Respiratorias (CIBERES CB06/06/0058), Palma de Mallorca, Islas Baleares, Spain; Beijing Institute of Microbiology and Epidemiology, China

## Abstract

Microbiological confirmation of a urinary tract infection (UTI) takes 24–48 h. In the meantime, patients are usually given empirical antibiotics, sometimes inappropriately. We assessed the feasibility of sequentially performing a Gram stain and MALDI-TOF MS mass spectrometry (MS) on urine samples to anticipate clinically useful information. In May-June 2012, we randomly selected 1000 urine samples from patients with suspected UTI. All were Gram stained and those yielding bacteria of a single morphotype were processed for MALDI-TOF MS. Our sequential algorithm was correlated with the standard semiquantitative urine culture result as follows: Match, the information provided was anticipative of culture result; Minor error, the information provided was partially anticipative of culture result; Major error, the information provided was incorrect, potentially leading to inappropriate changes in antimicrobial therapy. A positive culture was obtained in 242/1000 samples. The Gram stain revealed a single morphotype in 207 samples, which were subjected to MALDI-TOF MS. The diagnostic performance of the Gram stain was: sensitivity (Se) 81.3%, specificity (Sp) 93.2%, positive predictive value (PPV) 81.3%, negative predictive value (NPV) 93.2%, positive likelihood ratio (+LR) 11.91, negative likelihood ratio (−LR) 0.20 and accuracy 90.0% while that of MALDI-TOF MS was: Se 79.2%, Sp 73.5, +LR 2.99, −LR 0.28 and accuracy 78.3%. The use of both techniques provided information anticipative of the culture result in 82.7% of cases, information with minor errors in 13.4% and information with major errors in 3.9%. Results were available within 1 h. Our serial algorithm provided information that was consistent or showed minor errors for 96.1% of urine samples from patients with suspected UTI. The clinical impacts of this rapid UTI diagnosis strategy need to be assessed through indicators of adequacy of treatment such as a reduced time to appropriate empirical treatment or earlier withdrawal of unnecessary antibiotics.

## Introduction

Urinary tract infections (UTI) are among the most common infections [1]. Microbiological confirmation of a UTI takes 24–48 h. In the meantime, patients are usually given empirical antimicrobial therapy, sometimes unnecessarily or inadequately [2]. Anticipation of clinically useful information is of the utmost importance, with both diagnostic and therapeutic consequences [3], [4].

Traditionally, a rapid diagnosis of UTI entailed a Gram stain on urine samples [5]. Several studies conducted mostly in the 1970s and 1980s assessed the usefulness of this stain, which proved to be one of the most rapid, reliable and inexpensive methods for anticipating bacteriuria at >10^5^ colony forming units/ml [6]. However, the Gram stain has been abandoned as a routine diagnostic test in most microbiology laboratories.

A new technology, Matrix-Assisted Laser Desorption Ionization-Time Of Flight Mass Spectrometry (MALDI-TOF MS), has been recently introduced for the analysis of different biomolecules. This mass spectrometry procedure has been successfully used for the rapid identification of microorganisms already isolated by culture [7], [8] but has been scarcely employed for diagnostic purposes directly on clinical samples, with the exception of positive blood cultures, and urine samples [9]–[15].

In this article, we assess the feasibility of systematically performing a Gram-stain followed by MALDI-TOF MS on urine samples and determine the capacity of this algorithm to anticipate clinically useful information. This algorithm predicted the presence or absence of bacteriuria and the causative pathogen.

## Methods

### Ethics Statement

The study protocol received institutional review board approval by the “Comité Ético de Investigación Clínica Hospital General Universitario Gregorio Marañón” and the need for informed consent was waived.

### Setting

Our institution is a large teaching hospital with 1,550 beds that serves approximately 715,000 of Madrid’s inhabitants. It has an active surgery program including solid organ transplants.

### Processing of Urine Samples

During May-June 2012, we randomly selected 1000 urine samples from patients with UTI symptoms (minimal volume of urine submitted was 15 ml). All samples were cultured (2.5 µl) on Cystine Lactose-Electrolyte-Deficient (CLED) agar (bio-Mérieux, Marcy l’Etoile, France) and incubated in ambient air at 35°C for 18 h. Negative plates were incubated for a further 24 h. The cutoff for a positive culture was ≥10^5^ colony forming units (cfu) per milliliter of one or two different species [16]. Identification and susceptibility testing were performed by conventional methods (MicroScan^©^, Siemens, Tarrytown, NY).

### Gram Stain

A Gram stain was performed on all uncentrifuged samples and those with bacteria of a single morphotype per 20 oil-immersion fields were processed for MALDI-TOF MS spectrometry.

### Gram Stain Interpretation

Agreement between the Gram stain and the culture result was defined as follows.

Full agreement: the Gram stain result was consistent with the bacteria recovered in the culture. Partial agreement: only one of two microorganisms observed was recovered in culture, or viceversa, one of two microorganisms recovered in the culture was not observed. Disagreement: microorganisms observed were not recovered in culture and/or those not observed did appear on the culture plates.

### MALDI-TOF Mass Spectrometry

- Sample preparation. Each 15 ml urine sample was centrifuged at 1,500 revolutions per minute (rpm) for 10 min to remove leukocytes. The supernatant was collected and aliquoted into 1.5 ml-microcentrifuge tubes and centrifuged at 13,000 rpm for 2 min. Pellets from all tubes were recovered and resuspended in 1 ml high-performance liquid chromatography (HPLC)-quality water (Sigma-Aldrich, St. Gallen Rheintal, Switzerland). After another centrifugation at 13,000 for 1 min, the pellet was incubated in 0.2% Tween-80 (Sigma-Aldrich) for 5 min and centrifuged at 13,000 rpm for 1 min. The pellet was then washed to eliminate the detergent and centrifuged at 13,000 rpm for a further minute.

After discarding the supernatant, the pellet was spotted onto a polished steel MALDI target plate using a 1 µl sterile loop and allowed to dry. Next, it was covered with 1 µl of formic acid (70% vol/vol) and left to dry. Then, 1 µl of matrix solution (α-cyano-4-hydroxy-cinnamic acid solution in 50% acetonitrile and 2.5% trifluoroacetic acid) was added prior to the acquisition of spectra in a mass spectrometer.

Samples in which no microorganism identification was possible were further tested after a protein extraction step (see supplemental material). All extracted and non-extracted samples were tested in duplicate to determine the reproducibility of the method. The final score for each sample was the average score of the two spots. The turnaround time was 30 min for the first method and 45 min for the second.

- MALDI-TOF MS. Measurements were acquired in a Microflex LT bench top mass spectrometer (Bruker Daltonics, Germany) using the default settings. According to the manufacturer, a score ≥2.0 indicated species identification, a score between 1.7 and 2.0 indicated genus identification and a score of <1.7 indicates no identification.
- MALDI-TOF MS interpretation. The MALDI-TOF MS result was correlated with the culture result as follows: *true positive*, the MALDI-TOF MS and culture result were consistent; MALDI-TOF MS identified only one microorganism when two different microorganisms were isolated in the culture; in patients with a recent UTI diagnosis, on antibiotic treatment and with a negative urine culture, MALDI-TOF MS identified a microorganism concordant with the prior positive urine culture; *true negative*, a negative culture in the absence of MALDI-TOF MS identification; *false positive*, MALDI-TOF MS identification differed from the microorganism(s) identified in the cultures; or *false negative*, a positive culture in the absence of MALDI-TOF MS identification.

### Sequential Algorithm (Gram Stain Followed by MALDI-TOF MS) Interpretation

Our algorithm was correlated with the culture result, as follows:

Match: the information that would be reported to the clinician according to the Gram stain ± MALDI-TOF MS results was anticipative of the culture result. We defined as a match any of the following situations:

- No microorganisms detected by Gram staining (and therefore no MALDI-TOF MS was performed) in samples returning a negative culture;
- Microorganisms detected by Gram staining and MALDI-TOF MS identification matched the microorganism(s) identified in samples;
- Mixed flora detected by Gram staining (and therefore no MALDI-TOF was performed) in samples returning a contaminated culture.

Minor error: the information that would be reported to the clinician according to the Gram stain ± MALDI-TOF MS result was partially anticipative of the culture result. We defined as a minor error any of the following situations:

- No microorganisms detected by Gram staining (and therefore no MALDI-TOF was performed) in samples returning a contaminated culture;
- Mixed flora detected by Gram staining (and therefore no MALDI-TOF was performed) in samples returning a negative or positive culture;
- Microorganisms detected by Gram staining in the absence of MALDI-TOF MS identification in samples returning a negative or a matching positive culture.

Major error: the information that would be reported to the clinician according to the Gram stain ± MALDI-TOF MS result was incorrect and would have potentially led to inappropriate changes in antimicrobial therapy. We defined as major error any of the following situations:

- No microorganisms detected by Gram staining (and therefore no MALDI-TOF was performed) in samples returning a positive culture.
- Microorganisms detected by Gram staining and MALDI-TOF MS identification did not match the culture result.

### Statistics

Categorical variables are provided with their frequency distributions. Continuous variables are summarized as medians and inter-quartile ranges (IQR). For the Gram stain, mass spectrometry and sequential algorithm methods, sensitivity, specificity, positive and negative predictive values (PPV and NPV only for the Gram stain), and positive (+LR) and negative (−LR) likelihood ratios, with their 95% confidence intervals, were calculated. Accuracy was defined as the sum of true positive and true negative results. All statistical tests were performed using SPSS ver. 15.0.

## Results

Over the study period, 1000 samples from 958 patients were randomly selected for inclusion in the study: 43.2% of these patients were adult men (n = 414), and 3.8% were under 18 years of age (n = 36). Median age was 60.2 years (IQR 41.2–76.3) with no differences between sexes. Distribution by sampling technique was: 91.6% midstream voided, 6.8% bladder catheterization, 1.1% obtained during surgery and 0.6% obtained from patients with a permanent urinary catheter. The origin of the samples was: inpatients, 42.1%; emergency department, 28.8%; outpatient clinics, 25.8%; primary care, 2.6%; unknown, 0.7%.

Of the 1000 samples examined, 242 returned a positive culture. Out of 252 microorganisms isolated, 208 (82.5%) were Gram-negative, 36 (14.3%) were Gram-positive and 8 (3.2%) were yeasts. In only 10 samples (4.1%) were two different microorganisms isolated. The most frequently isolated microorganisms were *Escherichia coli* (58.3%), *Enterococcus faecalis* (8.3%) and *Klebsiella pneumoniae* (7.9%).

### Gram Stain

All 1000 samples were Gram stained. Microorganisms were detected in 267 samples. Gram negative microorganisms were seen in 52.1% of the occasions, Gram positive microorganisms in 20.2%, yeasts in 5.2% and mixed flora in 22.5%. Out of 733 samples in which no microorganisms were detected by Gram staining, 93.2% (n = 683) returned negative cultures.

Full agreement of the Gram stain with the culture result was recorded for 854 or 85.4% of the samples (95% CI 83.2–87.6); partial agreement for 46 or 4.6% (95% CI 3.3–5.9) and disagreement for 100 or 10.0% (95% CI 8.1–11.9). To define the diagnostic performance of the Gram stain, “partial agreements” were considered as true positive results. The diagnostic performance of the Gram stain was: Se 81.3% (95% CI 76.4–86.1), Sp 93.2% (95% CI 91.3–95.1), PPV 81.3% (95% CI 76.4–86.1), NPV 93.2% (95% CI 91.3–95.1), +LR 11.91 (95% CI 9.06–15.67), −LR 0.20 (95% CI 0.16–0.26) and accuracy 90.0% (95% CI 88.1–91.9).

### MALDI-TOF MS

The 207 samples yielding a single morphotype in the Gram stain were subjected to MALDI-TOF MS. The performance of this test was as follows: correct identification, 130, and only one microorganism identified, 7 (true positives), for a total of 137 (66.2%); no identification in samples with a negative culture (true negative), 25 (12.1%); incorrect identification (false positive), 9 (4.3%); and no identification in samples returning a positive culture (false negative), 36 (17.4%).

The protein extraction protocol was employed every time MALDI-TOF MS failed to offer an identification. That is, it was used on 84 out of 207 samples (40.6%). This procedure allowed to reach a final identification in 20/207 (9.7%) additional samples and gave 3 false positive results. Without extraction, MALDI-TOF’s diagnostic performance was: Se 68.0% (95% CI 60.8–75.2), Sp 81.3 (95% IC 66.2–96.3), +LR 3.63 (IC95% 1.75–7.51), and −LR 0.39 (95% CI 0.30–0.52). With extraction, MALDI-TOF’s diagnostic performance was: Se 79.2% (95% CI 72.9–85.5), Sp 73.5 (95% IC 57.2–89.8), +LR 2.99 (IC95% 1.70–5.27), and −LR 0.28 (95% CI 0.20–0.40). Thus, without extraction, sensitivity would have been lower.

In our study, out of 1000 urine samples, 10 were found to have 2 different species in cultures; MALDI-TOF MS identified 1 of the 2 isolates in two samples, provided an unreliable identification in one sample and provided no identification in the remaining 7 samples.

In Table 1 we present data on MALDI-TOF MS and conventional identification of monomicrobial cultures in patients with UTI.

**Table 1:** MALDI-TOF MS versus conventional identification of 242 positive monobacterial cultures.

Conventional identification (no. of cases)	Correlation with Gram stain (%)	Correlation with MALDI-TOF MS identification (%)	MALDI-TOF MS score (median, IQR)*
*Escherichia coli* (144)	84.0	84.2	2.1 (2.0–2.2)
*Klebsiella pneumoniae* (20)	90.0	75.0	2.2 (2.0–2.3)
*Enterococcus faecalis* (19)	78.9	60.0	1.9 (1.8–2.1)
*Pseudomonas aeruginosa* (9)	77.7	60.0	2.1 (1.8–2.2)
*Proteus mirabilis* (7)	85.7	80.0	2.2 (2.1–2.2)
*Candida albicans* (6)	66.7	–	–
*Staphylococcus aureus* (5)	80.0	75.0	1.8 (1.7–2.3)
*Enterobacter cloacae* (4)	75.0	100.0	2.0 (1.8–2.2)
*Candida glabrata* (3)	100.0	100.0	1.7 (1.7–1.8)
*Klebsiella oxytoca* (3)	66.7	50.0	2.4
*Acinetobacter baumanii* (2)	100.0	100.0	1.8 (1.8–1.9)
*Enterococcus faecium* (2)	50.0	–	–
*Lactobacillus* spp. (2)	100.0	100.0	1.9 (1.8–1.9)
*Morganella morganii* (2)	100.0	100.0	2.0 (1.7–2.4)
*Staphylococcus epidermidis* (2)	100.0	100.0	1.8 (1.7–2.0)
*Streptococcus agalactiae* (2)	50.0	100.0	1.7
*Streptococcus gallolyticus* (2)	50.0	100.0	1.7
*Candida krusei* (1)	100.0	–	–
*Candida tropicalis* (1)	100.0	–	–
*Enterobacter sakazakii* (1)	100.0	100.0	1.9
*Gardnerella vaginalis* (1)	100.0	100.0	1.7
*Providencia stuartii* (1)	100.0	–	–
*Pseudomonas putida* (1)	–	Not performed	–
*Serratia marcescens* (1)	–	Not performed	–
*Streptococcus viridans* (1)	–	Not performed	–

* When there is only one bacterial strain, the corresponding MALDI-TOF MS score is given.

False positive MALDI-TOF MS results did not match the Gram stain results. True negative MALDI-TOF MS identifications corresponded to false positive Gram stain results. False negative MALDI-TOF MS results did not correspond to any particular bacterial species.

We did not find any differences in the performance of MALDI-TOF MS with the various types of organisms causing UTI. Nevertheless, this could be due to small sample size.

### Sequential Testing Algorithm

The combined performance of the two techniques is shown in Table 2. Overall, our testing algorithm would have provided clinicians with information anticipative of the culture result (82.7%, 95% CI 80.3–85.1) or with minor errors (13.4%, 95% CI 11.2–15.6) for 96.1% of the samples. Only for 3.9% (95% CI 2.7–5.1) of the samples, would the information provided to clinicians have potentially led to changes in antimicrobial therapy that would not benefit the patient.

**Table 2:** Results of the sequential testing algorithm (Gram followed by MALDI-TOF MS).

Gram stain	MALDI-TOF MS	Culture result	Match n (%)	Minor error n (%)	Major error n (%)
No m.o. (n = 733)	Not performed	Negative	683 (68.3)	–	–
		Contaminated	–	12 (1.2)	–
		Positive	–	–	38 (3.8)
M.o.* seen (n = 207)	Correct identification	Negative**	1 (0.1)	–	–
		Contaminated***	7 (0.7)	–	–
		Positive	129 (12.9)	–	–
	Incorrect identification	Negative†	–	7 (0.7)	–
		Contaminated	–	1 (0.1)	–
		Positive	–	–	1 (0.1)
	No identification	Negative	–	25 (2.5)	–
		Positive††	–	36 (3.6)	–
Mixed flora(n = 60)	Not performed	Negative	–	15 (1.5)	–
		Contaminated	7 (0.7)	–	–
		Positive	–	38 (3.8)	–
Total (n = 1000)			827 (82.7)	134 (13.4)	39 (3.9)

* M.o.: microorganisms.

** Patient with a recent UTI diagnosis on antibiotic treatment. The UTI was caused by the *same* organism identified with MALDI-TOF MS, according to the prior positive urine culture.

*** One microorganism identified by MALDI-TOF MS.

† Patients with a recent UTI diagnosis on antibiotic treatment. The UTI was caused by a *different* organism than that identified with MALDI-TOF MS, according to the prior positive urine culture.

†† In 31/36 samples, α-defensins suppressed bacterial peaks rendering no identification with MALDI-TOF MS.

Of 207 samples in which a single morphotype was detected by Gram staining, MALDI-TOF MS provided an identification in 146 samples and provided no identification in 61 samples. When MALDI-TOF MS provided an identification, this was correct in 93.8% (137/146) of cases, whereas when no identification was given, this negative result was erroneous for 59.0% (36/61) of the samples.

When we examined the spectra recorded in 31/36 positive samples for which no MALDI-TOF MS identification was achieved, three intense peaks were observed, corresponding to the human α-defensins 1, 2 and 3, at a mass to charge ratio of around 3440 Da. The presence of these molecules is known to suppress bacterial protein peaks and therefore prevent database matching [14], acting as a confounder that precludes the etiologic diagnosis of UTI by MALDI-TOF MS performed on urine samples. All our samples containing defensins (n = 31) yielded a positive culture.

In summary, when a urine sample in which a single morphotype was detected by Gram staining returned a positive MALDI-TOF MS result, the likelihood of this result being correct (as established by the subsequent culture result) was 93.8%. When MALDI-TOF MS provided no result but peaks corresponding to human α-defensins were observed in the MALDI-TOF MS spectrum, a positive culture was obtained the following day. However, when MALDI-TOF MS provided no result but no such peaks were detected, in 25/30 of the cases the subsequent cultures were negative.

### Turnaround Time

**Figure 1:**
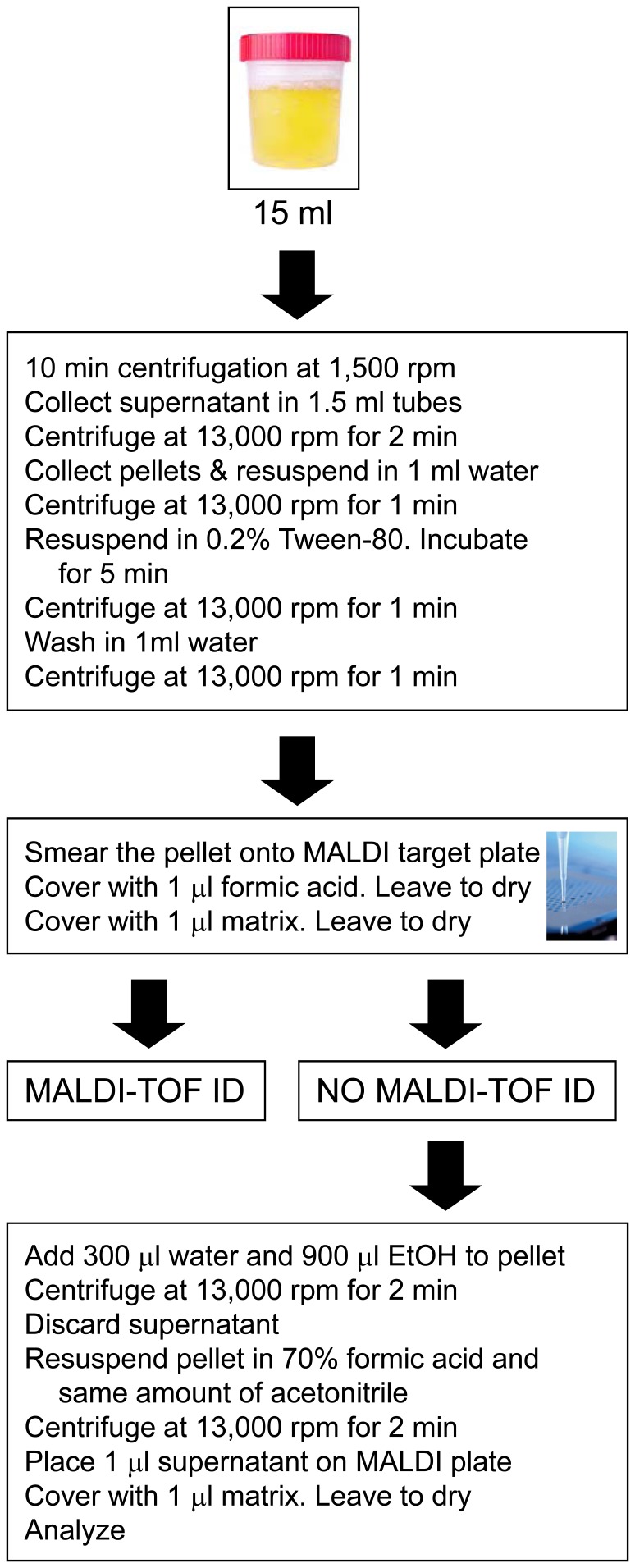
MALDI-TOF MS protocol used on urine samples.

The time needed for these two rapid techniques was significantly shorter than that required for the more conventional culture methods. For all samples, results were available within one h. We have included in Fig. 1 a flowchart describing the diagnostic procedure.

## Discussion

By performing a Gram stain followed by MALDI-TOF MS directly on urine samples we were able to predict the presence or absence of bacteriuria and the causative microorganism in patients with a suspicion of UTI reasonably well and within a working laboratory shift. According to our findings, physicians would receive therapeutically helpful information for 96.1% of samples in less than 1 h. Only in 0.4 out of 10 cases would inappropriate information be provided.

Managing patients with a UTI entails a considerable amount of work for clinicians and microbiology laboratory personnel [17]. The standard quantitative urine culture takes 2–3 days, such that before results are available, clinicians often initiate treatment with antibiotics. These antibiotics are in many cases unnecessary, inefficient or cover a wider spectrum of microorganisms than necessary [18]. The use of numerous tests to speed up the diagnosis [19]–[22] has been effectively assessed, but the performance of these tests (sensitivity/specificity) has been variable at: nitrite (41–64%/85–98%), leukocyte esterase (48–86%/17–93%), white-cell count in urine (67–80%/82–90%). Even when combined, false negative results occur in around 10% of patients with a UTI. Moreover, they do not assess the etiology of UTI and cannot replace urine cultures.

The usefulness of Gram staining uncentrifuged urine to identify significant bacteriuria was first demonstrated in 1968 [23] and this has since been occasionally used as a screening test for UTI [24]–[26]. Its reported accuracy for the diagnosis of UTI has been: sensitivity, 82.2–97.9%; specificity, 66.0–95.0%; positive predictive value, 31.6–94.3%, and negative predictive value, 95.2–99.5%, varying with the different counts of microorganisms in the sample, amongst other factors [25]–[28]. When compared to alternative rapid screening tests, the Gram stain has proved to be more accurate [22], [26] and less expensive [28] than other rapid screening tests. In addition, the time needed for Gram staining and microscopy examination is relatively short [5].

Some authors propose that only counts ≥10^5^ cfu/ml should be reported, as these are 73.9 times more likely to correlate with a clinically significant UTI than lower counts [29]. With this cutoff for UTI, only 38 positive urine samples were missed.

The clinical impacts of rapidly reporting Gram stain information on urine samples submitted for culture have not been sufficiently addressed and only one previous study including 57 samples has examined this issue to date [30].

The general consensus is that MALDI-TOF MS is a fast, reliable and cost-effective technique that is easily implemented [7], [8]. However, only a few studies have explored its use directly on organic fluids, before microbial isolation [31], [32]. Limited data related to the direct use of MALDI-TOF MS on urine samples are available [12]–[15]. One of the difficulties of this technique arises from insufficient numbers of bacteria in the sample; the threshold of microorganisms required varies among the different species [12], [15], [33], [34]. Thus, samples need to be preselected depending on their bacterial load [15]. We believe that the best way to do this is by Gram staining, which will also indicate the type of responsible microorganism.

Some authors have warned of the need to improve the sensitivity of this technology, although they admitted that the majority of urine samples collected from patients with common urinary tract infections have bacterial counts >10^5^ cfu/ml and can therefore be identified by MALDI-TOF MS [15].

UTI with counts under 10^5^ cfu/ml do, of course, exist. Present rapid screening tests (Gram stain, dipstick urine analysis or flow cytometry) are not sensitive enough for samples with bacterial counts under this threshold [35]. However, they have a high negative predictive value and this is why these screening methods should continued to be used to rule out bacteriuria and avoid plating negative urine samples, as are the majority of samples. The high costs and labor requirements of culture can only be controlled by effective utilization of this test [35], [36].

In patients with upper or complicated UTI, if there is no clinical improvement within 48 hours of starting antibiotic treatment, in the case of recurrent UTI, or if there are any other complicating factors, then no pre-screening should be done and these urine samples should all be cultured, giving value to counts ≥10^2–3^ cfu/ml.

There is some concern regarding the performance of MALDI-TOF MS directly on clinical samples testing positive for specific microorganisms. It is well known that yeast identification at the species level achievable by MALDI-TOF MS in positive blood cultures, even after protein extraction, is poor [9], [37]. The same can be said for certain streptococci [38]. This poor performance was also observed in our study. The scores obtained for urine cultures containing *Candida glabrata* using our diagnostic algorithm only support an identification at the genus level, which is not more informative for the clinician than the Gram stain. The same applied to urine cultures positive for certain Gram positive microorganisms such as *Streptococcus agalactiae* and *Streptococcus gallolyticus*. Still, for the Bruker MALDI Biotyper system, lowering the species-secure score cutoff to >1.7 instead of the manufacturer’s recommendation of ≥2.0 is now deemed acceptable by some authors [39], [40].

Regarding mixed urine cultures, in a recent study by Wang et al., the MALDI-TOF MS detection of 2 microorganisms present in urine specimens was tested by spiking sterile saline aliquots with 2 different bacterial species at different dilutions. The authors observed that the two types of bacterium could be identified in a mixture only if they appeared at ratios of 1∶1 or 1∶2 [15]. In their study, 2 different species were recovered from cultures in 44 urine samples (out of 1456 clinical samples). In 4 of these samples, 2 types of bacterium were identified by MALDI-TOF MS. In 20 samples, only 1 of the 2 isolates was identified and in the remaining 20 samples, MALDI-TOF MS provided an unreliable identification. Our experience was similar. These two examples indicate that this technology is not yet adequate for the identification of different bacterial strains directly on clinical samples. Still, as confirmed in our study, most UTI (over 90%) are monomicrobial [27], [41], [42].

In a study by Köhling et al., 107 urine samples from patients with clinical suspicion of UTI that were not preselected *a priori* for high bacterial counts revealed an overall sensitivity of the MALDI-TOF MS method of 60.7% [14]. These authors reported an interesting phenomenon. Thus, when they analyzed the spectra yielded by 22/26 positive samples for which no identification was achieved, they found a triplet of intense peaks corresponding to human α-defensins 1, 2 and 3, located at a mass to charge ratio of around 3440 Da. The presence of α-defensins suppressed bacterial peaks and prevented database matching such that microorganisms could not be identified directly in the urine samples. We also observed this phenomenon in our work. When the authors tested samples without defensins, the sensitivity of the MALDI-TOF MS procedure used directly on urine samples was 97.1% for counts ≥10^5^ cfu/ml.

In our study, after reviewing the medical records of the patients with human α-defensins, we could not find any common clinical feature among them with regard to type of patient, underlying disease, clinical presentation or outcome.

The potential clinical impacts of being able to identify the causative pathogen of any infection is directly related to how much knowing the taxon would serve to direct empiric antibiotic therapy [3]. To assess this, it is also desirable to know the local epidemiology (community –preferable– or hospital surveillance data) and associated resistance patterns in each setting [43]. MALDI-TOF MS has already proved its usefulness in reducing the time to effective and optimal antibiotic treatment in both bacteremia and candidemia [11], [44], [45]. In effect, the value of microbiologic data is often inversely proportional to the time interval necessary for its generation [3].

Our serial diagnostic algorithm Gram stain plus MALDI-TOF MS was able to provide in under an hour an etiologic diagnosis of UTI. This diagnosis allows for the adjustment of empiric antibiotic treatment, especially when the causative microorganism are not those most frequently associated with this type of infection (i.e., non-fermenting Gram-negative bacilli, yeasts) or when they have resistance mechanisms (i.e., AmpC betalactamase-producing *Enterobacteriaceae*, *Staphylococcus aureus*).

As a limitation of our study, we should mention that being a single-center study, its results may only reflect local practice patterns. However, we included 1000 urine samples from all types of patients and did not preselect any subgroup with greater clinical suspicion of UTI. A disadvantage of the method proposed is that it requires a minimum sample volume of 15 ml of urine.

Future studies should address the clinical impacts of our rapid diagnosis algorithm for UTI by examining its capacity to improve the adequacy of treatment in terms of reducing the time of empiric antibiotic treatment or allowing for the earlier withdrawal of unnecessary antibiotics.
